# Neutrophil/lymphocyte ratio elevation in renal dysfunction is caused by distortion of leukocyte hematopoiesis in bone marrow

**DOI:** 10.1080/0886022X.2019.1597736

**Published:** 2019-04-24

**Authors:** Satoyasu Ito, Yoshiya Ohno, Toshiyuki Tanaka, Shuhei Kobuchi, Kazuhide Ayajiki, Eri Manabe, Tohru Masuyama, Sakamoto Jun-Ichi, Takeshi Tsujino

**Affiliations:** a Division of Pharmaceutical Therapeutics, Department of Pharmacy, School of Pharmacy, Hyogo University of Health Sciences, Kobe, Japan;; b Division of Immunobiology, Department of Pharmacy, School of Pharmacy, Hyogo University of Health Sciences, Kobe, Japan;; c Division of Pharmacology, Department of Pharmacy, School of Pharmacy, Hyogo University of Health Sciences, Kobe, Japan;; d Cardiovascular Division, Department of Internal Medicine, Hyogo College of Medicine, Nishinomiya, Japan;; e Graduate School of Pharmacy, Hyogo University of Health Sciences, Kobe, Japan

**Keywords:** Neutrophil/lymphocyte ratio, chronic kidney disease, distortion of the leukocyte hematopoiesis process, bone marrow, AST-120

## Abstract

**Objective:** We investigate the mechanism of neutrophil/lymphocyte ratio (NLR) elevation, a useful prognostic marker in patients with cardiovascular diseases (CVDs).

**Methods:** In this clinical study, we retrospectively searched for factors associated with NLR elevation in cardiovascular outpatients. In animal experiments using mice with adenine-induced nephropathy, we further examined the hematopoietic process in bone marrow and explored the mechanism of NLR elevation.

**Result:** In patients with CVDs or their risk factors, multiple regression analysis revealed that decrease in estimated glemerular filtration rate and increase in white blood cell count were significantly associated with increase in NLR. In mice with adenine-induced nephropathy, NLR and serum indoxyl sulfate (IS) levels were increased. Fluorescence-activated cell sorting revealed the increase in the number of myeloid progenitors and decrease in the number of common lymphoid progenitors, suggesting biased granulocyte side in the hematopoietic process in bone marrow. Treatment with oral charcoal adsorbent AST-120 decreased serum concentration of IS and normalized NLR and bone marrow abnormalities in mice with adenine-induced nephropathy.

**Conclusion:** Renal function was a strong determinant of NLR in cardiovascular outpatients. NLR elevation due to renal impairment is caused by distortion of the hematopoietic process in bone marrow. IS plays a significant role in these processes.

## Introduction

In patients with chronic kidney disease (CKD), the morbidity and mortality of cardiovascular disease (CVD) such as heart failure, stroke, and myocardial infarction are increased. On the other hand, CVD is a major risk for CKD [1,2]. CVD and CKD form a vicious circle with each other and exert adverse effects, which is called cardio-renal syndrome [3]. Elucidation of molecular mechanisms of crosstalk between CKD and CVD is an important key for developing new treatment strategy of cardio-renal syndrome. Chronic inflammation has recently drawn attention as a new risk factor of CVD and CKD. Systemic inflammation is found in patients with CKD [4–6] and CVD [7–9]. Thus, chronic inflammation is suggested to be an important key that links CKD and CVD [10]. Neutrophil/lymphocyte ratio (NLR) has been reported as a useful marker of inflammation [11]. NLR has the advantage of low cost and widespread availability compared to other biomarkers of chronic inflammation. NLR has attracted attention since NLR is elevated in patients with various malignant tumors and CVD and a strong prognostic marker in them [12–16]. Although usefulness of NLR as predictor has being extensively studied, little research on the mechanisms by which NLR has been elevated. As NLR elevation is a disease condition common to various diseases, clarifying its mechanism may lead to the development of a new treatment method common to each disease.

Therefore, in the present study, we examined how renal function affects NLR in CVD. First, we clinically investigated what kind of clinical factors are related to NLR elevation in cardiovascular outpatients. Second, we investigated whether reduction of renal function causes NLR elevation in animal experiments. Third, we explored how indoxyl sulfate (IS), the most studied uremic substances among, alter NLR in CKD model mice.

## Materials and methods

### Clinical study

This study was approved by the Ethics Committee of Hyogo College of Medicine and the Ethics Committee of Hyogo University of Health Sciences.

We retrospectively investigated continuous outpatients who visited the Cardiovascular Division, Department of Internal Medicine, Hyogo College of Medicine Hospital from February 2011 to October 2012 for the treatment of CVD (ischemic heart disease, heart failure, peripheral artery disease, cerebrovascular disease, cardiomyopathy, and arrhythmia) or risk factors of CVD (hypertension, dyslipidemia, diabetes mellitus, and hyperuricemia), and who underwent complete blood cell counts, differential leukocyte count, and measurement of serum creatinine.

We reviewed patient demographics, comorbidity, and medication in their medical records. Exclusion criteria were as follows: history of digestive system surgery, malignancy, hemodialysis, aplastic anemia, hemolytic anemia, major bleeding within 1 year, inflammatory disease, connective tissue disease, and use of erythropoietin, vitamins, iron, or folic acid preparations. A Japanese equation was used to calculate estimated glomerular filtration rate (eGFR): eGFR (ml·min^−1^·1.73 m^−2^) =  194 × serum creatinine^−1.094^×age^−0.287^(×0.739 if female). Diabetes mellitus was defined as fasting plasma glucose ≥126 mg/dl, HbA1c ≥6.5%, or use of anti-diabetic medication. Hypertension was defined as systolic blood pressure ≥140 mmHg, diastolic blood pressure ≥90 mmHg, or use of anti-hypertensive medication. Dyslipidemia was defined as low-density lipoprotein cholesterol ≥140 mg/dl, high-density lipoprotein cholesterol <40 mg/dl, triglyceride ≥150 mg/dl, or use of anti-dyslipidemia medication. Anemia was defined according to the World Health Organization criteria [hemoglobin (Hb) <13 g/dl in men, <12 g/dl in women].

### Animal research

The study design was approved by the animal care and use committee of Hyogo University of Health Sciences based on the Guidelines for the Use of Laboratory Animals of Hyogo University of Health Sciences.

For the adenine-induced nephropathy model, 5 weeks-old balb/c mice were purchased from SLC (Shizuoka, Japan) and housed in standard housing conditions with unlimited access to food and water. Mice were divided into the following four groups (7–9 mice per group), namely the control group, the restricted-fed group, the adenine-fed group, and the adenine-fed + AST120 group. The mice in the control group were fed with powdered standard diet (Oriental Yeast Co., LTD. Tokyo, Japan). We fed the mice in the adenine-fed group and the adenine-fed + AST120 group with powdered standard diet added 0.7% adenine for 2 weeks to induce nephropathy. After adenine-diet was given for 2 weeks, mice in the adenine-fed group were given standard diet and bred for 2 weeks, and mice in the adenine-fed + AST120 group were administered powdered standard diet added 3 *w*/*w*% AST-120. It is known that body weight decreases due to adenine diet. To eliminate the effect of weight loss, the restricted-fed group was established. In the restricted-fed group, the mice were given the limited amount of powdered standard diet to be about the same body weight as mice in the adenine-fed group.

For the single indol loading model, 5 weeks-old balb/c mice were housed in standard housing conditions with unlimited access to food and water. After 20 h of starvation, we administrated indol (10 mg/kg; Nakalai Tesque: Kyoto, Japan) in 0.5% methyl cellulose (Wako Pure Chemical Industries) or vehicle by gavage. Ten hours after indicated treatment, mice were sacrificed and samples were obtained.

### Complete blood cell counts

A complete blood cell counts and differential leukocyte count was performed using Sysmex XT-2000i (Sysmex Kobe, Japan), which is optimized for mouse sample. Blood collected from the aorta was placed in a blood collection tube containing EDTA-2K. Samples were stored at room temperature until analysis.

### Biochemical and hematological parameters

Serum creatinine was measured by a commercially available kit (Lab Assay Creatinine, Wako Pure Chemical Industries Ltd., Osaka, Japan). Serum concentrations of IS were determined using HPLC [17].

## Materials

Charcoal adsorbent AST-120 (Kremezin^®^) was purchased from Mitsubishi Tanabe Pharma Co., Ltd. (Osaka, Japan). All other materials were purchased from Wako (Kyoto, Japan) or Nakalai Tesque (Kyoto, Japan) unless stated otherwise.

### Flow cytometry

Bone marrow cells were resuspended in cold phosphate-buffered saline. Flow cytometry was performed using BD FACS Aria™ II Instrument (BD Biosciences, San Diego, CA). Data were analyzed using Flow Jo software (FlowJo LLC, Ashland, OR). The following reagents (BioLegend, San Diego, CA) were used: anti-c-kit-APC, anti-IL-7Rα-FITC, anti-Sca-1-PE, anti-lineage-Biotin, streptavidin-PE/Cy7, and respective isotype controls.

### Statistical analysis

Data are given as mean ± SD for normally distributed continuous variables, median (interquartile range) for non-normally distributed continuous variables, or number (%) for categorical variables. Log-transformed NLR was used for statistical analysis because the distribution of NLR showed a right-skewed shape. Differences in continuous variables between two groups were assessed using unpaired Student’s *t*-test in clinical study. Kruskal–Wallis test with Steel–Dwass’s post-hoc analysis was used for comparison between multiple groups in animal research. Regarding continuous variables, the correlation between each variable was examined using Pearson’s correlation analysis. Multiple regression analysis was used to identify variables that might predict NLR. Factors with *p* < 0.1 on univariate analysis were selected as independent factors in multivariate analysis. Differences in categorical variables between two groups were assessed using Fisher’s exact test. Differences were considered statistically significant for *p* < 0.05. All calculations and analyses were performed using EZR (Saitama Medical Center, Jichi Medical University, Saitama, Japan), which is a graphical user interface for R (The R Foundation for Statistical Computing, Vienna, Austria). More precisely, it is a modified version of R commander designed to add statistical functions frequently used in biostatistics [18].

## Results

### Clinical research

We screened 634 patients, and 98 patients (age 70 ± 11, 58.2% male) were enrolled. Table 1 lists the clinical characteristics, comorbidities, and medications of study population.

**Table 1.** Patient clinical characteristics.

Clinical characteristics	
Age (years)	70 ± 11
Male gender	57 (58.2%)
Height (cm)	159.3 ± 8.6
Body weight (kg)	59.6 ± 11.9
Body mass index	23.5 ± 4.4
SBP (mmHg)	138.2 ± 23
DBP (mmHg)	78.6 ± 14.8
Heart rate (beats/min)	76.5 ± 15.3
WBC (×10^2^/μL)	61.6 ± 17.9
Neut (%)	63.2 ± 9.5
Lymph (%)	26.6 ± 8.9
Mono (%)	6.0 ± 1.8
Eosino (%)	3.6 ± 2.7
Baso (%)	0.6 ± 0.4
Neutrophil/lymphocyte	2.8 ± 1.5
Log-NLR	0.4 ± 0.2
Plt (×10^4^/μL)	20.3 ± 5.5
RBC (×10^4^/μL)	428 ± 67
Hb (g/dL)	13.0 ± 2.1
MCV (fL)	92.4 ± 4.9
RDW-CV	13.8 ± 1.1
eGFR (mL/min/1.73m^2^）	60.4 ± 27.1
Comorbidity	
Hypertension	74 (75.5%)
Diabetes mellitus	43 (43.9%)
Dyslipidemia	47 (48.0%)
Heart failure	18 (18.4%)
Angina pectoris	25 (25.5%)
Old myocardial infarction	22 (22.4%)
History of PCI	20 (20.4%)
Cerebral infarction	13 (13.3%)
Cerebral hemorrhage	2 (2.0%)
Peripheral artery disease	24 (24.5%)
History of gastroduodenal ulcer	7 (7.1%)
Reflux esophagitis	9 (9.2%)
Medication	
Antiplatelet agents	52 (53.1%)
ARB	51 (52.0%)
Calcium channel blockers	46 (46.9%)
β-blockers	38 (38.8%)
Diuretic agents	32 (32.7%)
Statins	30 (30.6%)
PPI	30 (30.6%)
Oral hypoglycemic agents	19 (19.4%)
H_2_-blockers	15 (15.3%)
Warfarin	13 (13.3%)
ACEI	11 (11.2%)
Uric acid-lowering agents	7 (7.1%)
Insulin	3 (3.1%)

Data given as mean ± SD or n (%). ACEI: angiotensin-converting enzyme inhibitor; ARB: angiotensin receptor blocker; DBP: diastolic blood pressure; eGFR: estimated glomerular filtration rate; H_2_-blocker: histamine type 2 receptor blocker; Hb: hemoglobin; Ht: hematocrit; MCV: mean corpuscular volume; log-NLR: log-transformed neutrophil/lymphocyte ratio; PCI: percutaneous coronary intervention; Plt: platelet; PPI: proton pump inhibitor; RBC: red blood cells; RDW-CV: coefficient of variation of red cell distribution width; SBP: systolic blood pressure; WBC: white blood cells.

In categorical variables, diabetes mellitus, cerebral infarction, peripheral artery disease, insulin use, ACE inhibitor use, and diuretics use were associated with elevated log-NLR (Table 2). In continuous variables, log-NLR was positively correlated with white blood cell (WBC) and coefficient of variation of red cell distribution width, and negatively correlated with Hb and eGFR (Table 3). Multiple regression analysis was performed with factors whose *p* values was less than 0.1 in univariate analysis. We found that decrease in eGFR and increase in WBC count were independently associated with increase in log-NLR (Table 4).

**Table 2.** Effects of comorbidities and medications on log-NLR.

	+	−	*p*-value
Male gender (M+,W-)	0.405 ± 0.214	0.385 ± 0.226	0.654
Comorbidity			
Hypertension	0.417 ± 0.211	0.333 ± 0.234	0.102
Diabetes mellitus	0.453 ± 0.221	0.353 ± 0.208	0.024
Dyslipidemia	0.372 ± 0.201	0.420 ± 0.233	0.280
Heart failure	0.452 ± 0.177	0.384 ± 0.226	0.234
Angina pectoris	0.389 ± 0.179	0.399 ± 0.231	0.839
Old myocardial infarction	0.406 ± 0.222	0.394 ± 0.218	0.824
History of PCI	0.420 ± 0.194	0.391 ± 0.225	0.602
Cerebral infarction	0.548 ± 0.242	0.374 ± 0.207	0.007
Cerebral hemorrhage	0.564 ± 0.188	0.393 ± 0.218	0.276
Peripheral artery disease	0.536 ± 0.217	0.352 ± 0.200	<0.001
History of gastroduodenal ulcer	0.459 ± 0.234	0.392 ± 0.218	0.439
Reflux esophagitis	0.466 ± 0.179	0.390 ± 0.222	0.320
Medications			
ARB	0.400 ± 0.194	0.394 ± 0.244	0.894
Diuretic agents	0.490 ± 0.211	0.351 ± 0.209	0.003
ACEI	0.531 ± 0.213	0.380 ± 0.214	0.030
Calcium channel blockers	0.436 ± 0.195	0.362 ± 0.234	0.095
β-blockers	0.389 ± 0.171	0.402 ± 0.245	0.770
Antiplatelet agents	0.426 ± 0.242	0.364 ± 0.190	0.166
Warfarin	0.426 ± 0.176	0.392 ± 0.225	0.605
PPI	0.415 ± 0.203	0.389 ± 0.226	0.584
H_2_-blockers	0.425 ± 0.241	0.392 ± 0.215	0.593
Statins	0.403 ± 0.201	0.394 ± 0.227	0.862
Uric acid-lowering agents	0.436 ± 0.249	0.394 ± 0.217	0.622
Oral hypoglycemic agents	0.420 ± 0.237	0.391 ± 0.215	0.603
Insulin	0.733 ± 0.094	0.386 ± 0.213	0.006

Data given as mean ± SD or n (%). Differences in continuous variables between two groups were assessed using unpaired Student’s *t*-test.

ACEI: angiotensin-converting enzyme inhibitor; ARB: angiotensin receptor blocker; H_2_-blocker: histamine type 2 receptor blocker; log-NLR: log-transformed neutrophil/lymphocyte ratio; PCI: percutaneous coronary intervention; PPI: proton pump inhibitor.

**Table 3.** Correlation between clinical parameters and log-NLR.

	R	*p*-value
Age	0.110	0.282
Height	0.112	0.279
Body weight	−0.010	0.921
Body mass index	−0.087	0.402
SBP	0.069	0.536
DBP	−0.024	0.832
Heart rate	0.135	0.252
WBC	0.323	0.001
Mono (%)	−0.149	0.143
Eosino	−0.007	0.947
Baso	−0.060	0.556
Plt	0.099	0.334
Hb	−0.315	0.002
MCV	−0.065	0.526
RDW-CV	0.281	0.005
eGFR	−0.524	<0.001

Univariate analysis of factors associated with NLR was examined by Pearson’s correlation analysis.

eGFR: estimated glomerular filtration rate; Hb: hemoglobin; Ht: hematocrit; MCV: mean corpuscular volume; log-NLR: log-transformed neutrophil/lymphocyte ratio; Plt: platelet; RDW-CV: coefficient of variation of red cell distribution width; SBP: systolic blood pressure; WBC: white blood cells.

**Table 4.** Multivariate analysis of factors associated with log-NLR.

	β (95% CI)	*p*-value
eGFR (ml·min^−1^·1.73 m^−2^)	−0.003 (−0.005 ∼ −0.001)	0.004
WBC (×10^2^/μL)	0.004 (0.001 ∼ 0.006)	0.004
Calcium channel blockers	0.058 (−0.019 ∼ 0.135)	0.137
Hb (g/dL)	−0.016 (−0.039 ∼ 0.007)	0.176
Cerebral infarction	0.077 (−0.038 ∼ 0.192)	0.185
ACEI	0.058 (−0.068 ∼ 0.184)	0.361
RDW-CV	0.014 (−0.026 ∼ 0.054)	0.496
Insulin	0.076 (−0.168 ∼ 0.320)	0.536
Peripheral artery disease	−0.032 (−0.156 ∼ 0.093)	0.617
Diabetes mellitus	−0.018 (−0.110 ∼ 0.074)	0.693
Diuretics	0.005 (−0.086 ∼ 0.096)	0.912

β: regression coefficient of multiple linear regression analysis; 95% CI: 95% confidence interval; ACEI: angiotensin-converting enzyme inhibitor; eGFR: estimated glomerular filtration rate; Hb: hemoglobin; log-NLR: log-transformed neutrophil/lymphocyte ratio; RDW-CV: coefficient of variation of red cell distribution width; WBC: white blood cells.

### Animal research

#### Body weights and BP

Body weight in the restricted-fed and adenine-fed groups significantly decreased compared to the control group. This change was ameliorated by AST-120 (Table 5). No significant difference was observed in body weight between the restricted-fed group and the adenine-fed group. Regarding blood pressure, there was no difference between the four groups.

**Table 5.** Complete blood cell counts and biochemical findings in mice.

	Ctrl.	res.	ade.	ade.+AST
Body weight [g]	24.5 (2.5)	19.1^a^ (1.8)	16.3^a^ (1.8)	23.0^abc^ (2.4)
SBP (mmHg)	110 (9)	102 (20)	107 (17)	107 (17)
creatinine [mg/dl]	1.7 (0.1)	1.7 (0.1)	2.2^a^ (0.5)	1.9 (0.4)
WBC (10^2^/μl)	41.8 (18.1)	16.0^acd^ (14.3)	35.1 (12.7)	25.8 (10.1)
RBC (10^4^/μl)	828 (106)	902 (83)	912 (92)	900(74)
Hb (g/dl)	13.6 (1.3)	14.2 (1.2)	13.5 (1.1)	13.7 (1.5)
Ht (%)	39.0 (2.2)	41.8 (5.4)	40.6 (3.0)	39.8 (5.6)
Platelet (10^3^/μl)	104.3 (11.2)	108.8 (11.2)	134.0^ab^ (30.4)	127.0 (15.5)
Reticulocyte	36.6 (10.5)	39.1 (16.9)	19.5 (37.9)	39.8 (7.4)

Data given as the variables in median (interquartile range).

The mice in the control group were fed with standard diet for 4 weeks. The mice in the restricted-fed group were given the limited amount of powdered standard diet. The mice in the adenine-fed group were fed with powdered adenine diet for 2 weeks, and after that, they were given standard diet. The mice in the adenine-fed + AST120 group were administered powdered standard diet added 3 w/w% AST-120.

ctrl: the control group; res: the restricted-fed group; ade.: the adenine-fed group; AST: the adenine-fed + AST120 group; Hb: hemoglobin; Ht: hematocrit; RBC: red blood cells; SBP: systolic blood pressure; WBC: white blood cells.

a *p* < 0.05 vs. the control group.

b *p* < 0.05 vs. the restricted-fed group.

c *p* < 0.05 vs. the adenine-fed group.

d *p* < 0.05 vs. the adenine-fed + AST120 group.

#### Complete blood cell counts and biochemical findings

**Figure 1.**
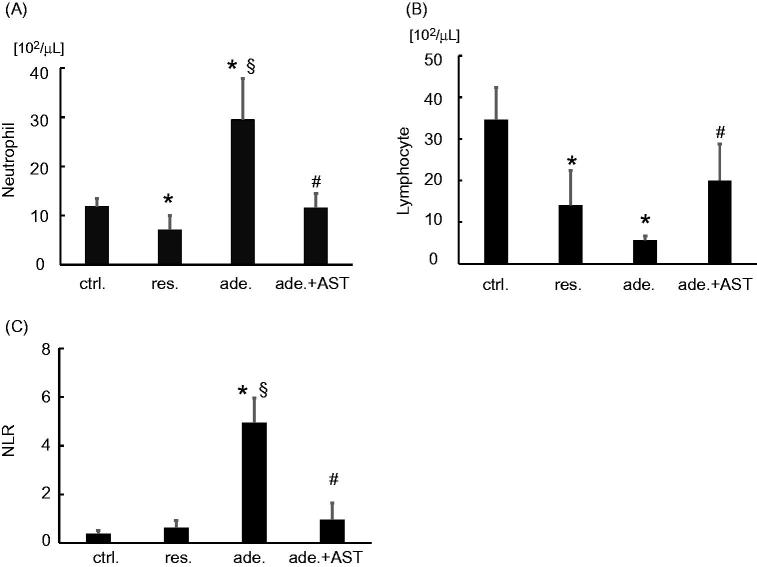
Differential count of leukocytes and neutrophil/lymphocyte ratio (NLR). The number of (A) neutrophils, (B) lymphocyte, and (C) log-NLR in the control group, the restricted-fed group, the adenine-fed group, and the adenine-fed + AST120 group, which are abbreviated as ctrl., res., ade., and ade.+ AST., respectively. *p < 0.005 vs. the control group; § *p* < 0.001 vs. the restricted-fed group. # *p* < 0.05 vs. the adenine-fed group.

WBC was significantly reduced in the restricted-fed group compared with the control, restricted-fed, and adenine-fed + AST120 groups (Table 5). Platelet was significantly increased in the adenine-fed group compared with the control and restricted-fed groups. There was no significant difference between the four groups in red blood cell, Hb, hematocrit (Ht), and reticulocyte (Ret). Serum creatinine levels were increased significantly in the adenine-fed group. Treatment with AST-120 tended to decrease the serum creatinine levels, which was not statistically significant (Table 5). The number of neutrophil decreased significantly in the restricted-fed group (*p* < 0.005) and markedly increased in the adenine-fed group (*p* < 0.001), compared with the control group (Figure 1(A)). The number of lymphocytes significantly decreased in the restricted-fed and the adenine-fed groups compared to the control group (*p* < 0.001) (Figure 1(B)). NLR significantly increased in the adenine-fed group compared with the control group and the restricted-fed group (*p* < 0.001). Treatment with AST-120 ameliorated the increase in the number of neutrophils, the decrease in the number of lymphocytes and increase in the NLR induced by adenine-feeding (Figure 1(C)).

**Figure 2.**
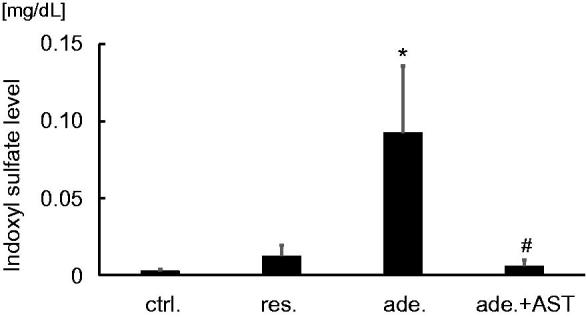
Serum levels of indoxyl sulfate (IS). Serum levels of IS in the control group, the restricted-fed group, the adenine-fed group, and the adenine-fed + AST120 group, which are abbreviated as ctrl., res., ade., and ade + AST., respectively. *p < 0.001 vs. the control group; #*p* < 0.001 vs. the adenine-fed group.

Figure 2 shows the serum levels of IS in the four groups. The serum IS level was significantly higher in the adenine-fed group than in the control group (*p* < 0.001), and treatment with AST-120 normalized it (*p* < 0.001).

**Figure 3.**
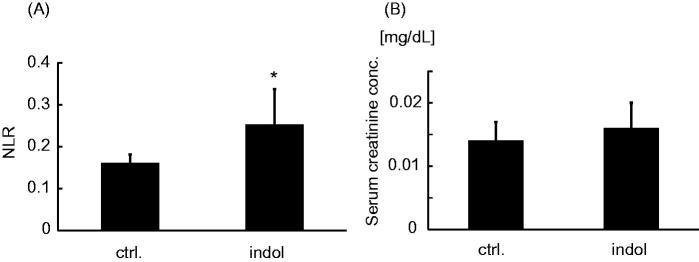
The influence of indol administration on neutrophil/lymphocyte ratio and renal function. (A) Neutrophil/lymphocyte ratio (NLR) and (B) serum creatinine concentration of the control group and the indol-administration group. The control group and the indol-administration group, which are abbreviated as ctrl. and indol, respectively. *p < 0.05 vs. the control group

Oral administration of indol significantly increased NLR in mice (Figure 3(A)). However, there was no significant difference between serum levels of creatinine in each group (Figure 3(B)).

#### Flow cytometry

**Figure 4.**
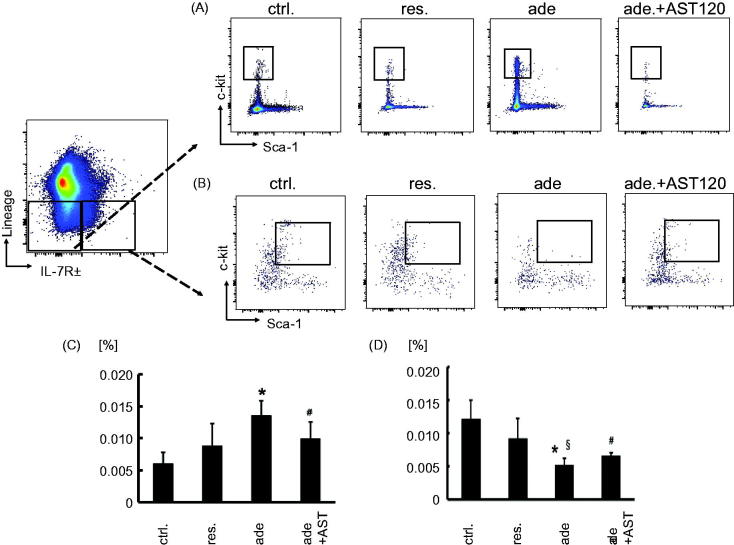
Myeloid progenitor cells and common lymphoid progenitor cells expression in bone marrow cells of in adenine-induced nephropathy. Gating for (A) myeloid progenitor cells and for (B) common lymphoid progenitor cells isolated from the control group, the restricted-fed group, the adenine-fed group, and the adenine-fed + AST120 group, which are abbreviated as ctrl., res., ade., and ade + AST., respectively. (C) The relative frequency of myeloid progenitor cells in bone marrow of each group. (D) The relative frequency of common lymphoid progenitor cells in bone marrow of each group. *p < 0.001 vs. the control group; #*p* < 0.05 vs. the adenine-fed group. §*p* < 0.05 vs. the restricted-fed group.

Figure 1 showed that the number of neutrophil increased and the number of lymphocyte decreased systemically in CKD model mice. Hematopoietic progenitors develop in bone marrow. So, we focused on myeloid and lymphoid hematopoietic progenitor cells, assumed that these changes are occurring in the hematopoiesis process in the bone marrow. We analyzed the development of myeloid progenitors (MP) (Lin- IL-7Rα- c-kit + Sca-1-) and common lymphoid progenitors (CLP) (Lin- IL-7Rα+ c-kit + Sca-1+) in bone marrow [19]. A significant increase in MP was observed in the adenine-fed group, and this change was ameliorated by the treatment with AST-120 (Figure 4(A,C)). In contrast to the expansion of MP, flow cytometric analyses revealed the reduction in the relative frequency in CLP, and this change was also ameliorated by the treatment with AST-120 (Figure 4(B,D)). The proportions of MP and CLP were not significantly changed by restricted-feeding from control (Figure 4(A–D)).

**Figure 5.**
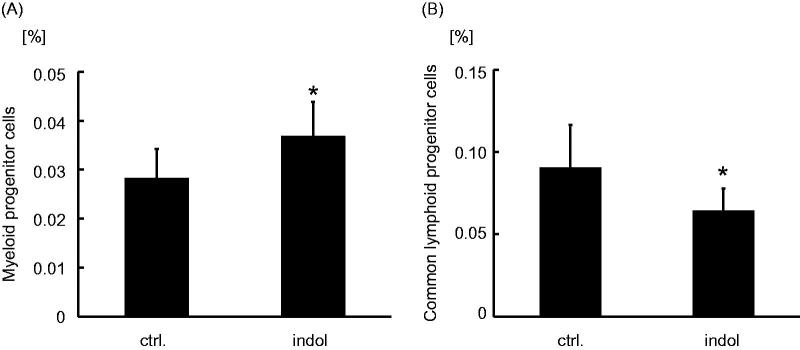
Myeloid progenitor cells and common lymphoid progenitor cells expression in bone marrow cells of indol-administered mice. The relative frequency of (A) myeloid progenitor cells and (B) common lymphoid progenitor cells in bone marrow of the control group and the indol-administration group, which are abbreviated as ctrl. and indol, respectively. *p < 0.001 vs. the control group.

Myeloid progenitors cells were increased in the indol-administration group compared with the control group (Figure 5(A)). On the other hand, CLP cells were decreased in the indol-administration group compared with the control group (Figure 5(B)).

## Discussion

NLR elevation has been reported to be a useful prognostic marker for various diseases [12,13]. In the present study, we investigated the mechanism of NLR elevation in cardiovascular outpatients. Our clinical research has revealed that reduced renal function and increased leukocytes were significantly associated with NLR elevation in patients with CVD or their risk factors. NLR elevation has been shown to be associated with CKD in patients with chronic heart failure [15]. In our study population, which was heterogeneous but reflected ‘real world’ of cardiovascular clinical setting, NLR elevation was associated with renal dysfunction. Thus, renal function affects NLR in a wide variety of patients with CVD. Further, animal experiments of the adenine-induced nephropathy have revealed that renal insufficiency was accompanied by increase in the number of MP and decrease in the CLP in bone marrow, and increased NLR in peripheral blood. These findings suggest that renal insufficiency induces NLR elevation by the distortion of the leukocyte hematopoiesis process in bone marrow.

We also examined the effect of oral charcoal adsorbent AST-120 on NLR elevation in these mice. AST-120 reduces serum IS concentration by adsorbing indol, a precursor of IS, in the intestine and suppresses absorption, thereby preventing progression of kidney damage [20]. In this study, AST-120 ameliorated elevation in serum IS, renal insufficiency, and the distortion of the leukocyte hematopoiesis process in the adenine-induced nephropathy. These findings suggest that IS plays a pivotal role in NLR elevation in CKD model mice.

It is thought that uremia symptoms are caused by uremic toxins accumulated in the body of renal disorder patients. IS is the most studied substance as a representative of uremic substances among about 90 uremic substances. In patients with CKD, the total mortality rate and the CVD mortality rate were high in the serum IS high-level group [21]. In other words, IS was involved in high morbidity and mortality of CVD in patients with CKD. IS has been reported to activate NADPH oxidase, induce reactive oxygen species (ROS), and promote CKD progression [22–25]. Oxidative stress is deeply involved in pathogenesis of inflammation [26–28], which accelerates the progression of CKD [28,29]. In order to examine how IS is involved in NLR elevation, we focused on leukocyte hematopoiesis in bone marrow.

In the inflammatory state, changes in leukocyte hematopoiesis induce hematopoietic stem cell to produce myeloid biased progenitor cells, resulting in increased granulocyte production and decreased lymphocyte production [30,31]. IS may induce distortion of the leukocyte hematopoiesis process in the bone marrow and NLR elevation in peripheral blood through chronic inflammation. Another possible mechanism by which IS induces NLR elevation is activation of sympathetic nervous system. Under conditions of chronic variable stress in mice, sympathetic nerve fibers have been shown to release surplus noradrenaline in bone marrow, leading to an increased output of neutrophils and inflammatory monocytes through the β3-adrenergic receptor [32]. Uremic toxins including IS stimulate the bulbospinal neurons in the rostral ventrolateral medulla, which plays a pivotal role in the regulation of peripheral sympathetic function [33]. IS may induce distortion of the leukocyte hematopoiesis process in the bone marrow and NLR elevation in peripheral blood through chronic sympathetic activation.

Is NLR elevation just a marker or a new therapeutic target of CKD? Regarding the role of neutrophils on CKD pathology augmented with increasing NLR, we are considering the involvement of neutrophil extracellular traps (NETs). NETs are a biological defense mechanism by neutrophils, releasing DNA out of the cell and trapping pathogenic bacteria [34,35]. NETs are caused by NETosis, a concept of new cell death other than apoptosis [36,37]. NETosis is aggressive cell death of neutrophils via ROS [36]. Excessive NET formation causes autoimmune or inflammatory lesions of various diseases and cause deleterious effects on the host, such as rheumatoid arthritis, systemic lupus erythematosus, vasculitis, inflammation, coronary artery disease [38–41]. Since inflammation is involved in the progression of kidney disease, NETs could be involved in the pathogenesis of CKD [34,42–44]. Furthermore, it is reported that NETs are correlated with NLR and neutrophil counts in patients with uremia [37]. These findings support the hypothesis that NETs are involved in the elevation of NLR. NLR could be a new therapeutic target in CKD. Further studies are necessary.

There are several limitations in this experiment. First, the number of patients is small in clinical study. Second, AST-120 decreased serum IS levels and ameliorated NLR elevation in animal study. Treatment of AST-120 tended to decrease serum creatinine levels, which was not statistically significant. We cannot exclude the possibility that AST-120 ameliorated NLR elevation partially through the improvement of renal function. However, IS should be involved in the process of NLR elevation because single indol loading increased NLR without impairing renal function. Third, the precise mechanism of NLR elevation induced by IS is still under investigation. We have shown only the elevation of MP and suppression of CLP in CKD model mice. However, we have clarified that the increase in NLR in CKD is caused by distortion in the hematopoietic process in the bone marrow, proving the significance of advancing the study focusing on bone marrow.

In conclusion, renal function was a strong determinant of NLR in cardiovascular outpatients. NLR elevation due to renal impairment is caused by distortion in the hematopoietic process in bone marrow. IS plays a significant role in these processes.
